# Investigating causes of increased morbidity and mortality within psychiatric patients - Somatic comorbidities of inpatients in a german psychiatric community hospital

**DOI:** 10.1192/j.eurpsy.2022.1161

**Published:** 2022-09-01

**Authors:** C. Theisen, C. Kieckhäfer, F. Röpcke, E. Meisenzahl

**Affiliations:** LVR-Klinikum Düsseldorf, Heinrich-Heine University Düsseldorf, Department Of Psychiatrie, Düsseldorf, Germany

**Keywords:** prevention, Somatic comorbidities

## Abstract

**Introduction:**

The co-occurrence of mental illness and somatic comorbidities is a major cause of increased morbidity and mortality within psychiatric patients, compared to the general population. This may be caused by an unhealthy lifestyle, side effects of psychotropic drugs and systematic barriers in healthcare provision. The underlying mechanisms remain under-investigated.

**Objectives:**

We systematically investigated relevant barriers and risk factors to the utilization of primary care among severe mentally ill outpatients.

**Methods:**

In a cross-sectional analysis, the psychiatric as well as somatic diagnoses of inpatients of a German psychiatric community hospital were identified. Furthermore, somatic and psychiatric medication, blood values (HBA1C) and sociodemographic data of the patients were analyzed. The frequencies of the somatic diagnoses were presented according to psychiatric diagnoses. By a Chi-Square goodness-of-Fit Test the distribution of somatic diagnoses and drug classes were verified according to the total cohort as well as for each psychiatric diagnosis and in respect to sex.

**Results:**

Our results provide an overview of common comorbidities with regard to the psychiatric diagnosis. The medication, in relation to the recorded somatic comorbidities, as well as the blood values, allow a conclusion to be drawn about the extent and success of the treatment.

**Conclusions:**

For the first time, real-life data on the somatic diagnoses and treatment of patients with a severe mental illness in a German hospital is presented. Our results will be used to create low-threshold interventions for the most relevant somatic comorbidities and to improve primary care of psychiatric patients through linking the care systems.

**Disclosure:**

No significant relationships.

